# Impact of light pollution spectra and intensity on the development and mortality of tiger mosquito (*Aedes albopictus*) larvae

**DOI:** 10.1242/jeb.252361

**Published:** 2026-09-17

**Authors:** Lauren E. Johnson, Emma R. Uder, Kathleen G. Dobbs, Elena Powell, Laura L. Tayon, Tom Radomski, Kim A. Medley, Katie M. Westby

**Affiliations:** ^1^ Tyson Research Center, Washington University in St Louis, Eureka, MO 63025, USA; ^2^ Department of Biology, Washington University in St Louis, St Louis, MO 63130, USA; ^3^ University of Notre Dame Environmental Research Center, Notre Dame, IN 46556, USA; ^4^ Northern Arizona University, Flagstaff, AZ 86011, USA

**Keywords:** Artificial light at night, ALAN, High-pressure sodium lamp, LED, Urban ecology, Urbanization, Emergence, Pupation, Mortality, Culicidae, Insect

## Abstract

Light pollution is a global urban stressor that can impact the behavior, physiology and fitness of many species, including disease vectors such as the tiger mosquito (*Aedes albopictus*). Experimental and observational studies in diverse arthropods have shown that artificial light at night (ALAN) can either slow development or accelerate it at the cost of higher mortality, suggesting that immature life stages may be vulnerable to light pollution. This study investigated how varying intensities of artificial light pollution – using both high-pressure sodium (HPS) and light-emitting diode (LED) lamps – affect the developmental rates of immature *Ae. albopictus* mosquitoes under semi-natural field conditions. During a controlled field experiment, newly hatched *Ae. albopictus* larvae were exposed to five different light intensity levels (10, 30, 60, 100 and 200 lx) under HPS and LED illumination alongside a control group without artificial light exposure. We measured time to pupation and emergence and mortality in both male and female mosquitoes to assess the biological impacts of varying light type and intensity. Our findings indicated no significant differences in average time to pupation or emergence across the different combinations of light type and intensity or control groups. However, greater variation was observed within certain light type and intensity combinations, suggesting potential nuances in ecological impacts not reflected in group averages. Mortality was consistently low across all groups, indicating that light pollution does not significantly impair larval survival in *Ae. albopictus*. These results suggest that under typical urban lighting conditions, *Ae. albopictus* development and immature survival remain robust.

## Teaser

**Summary:** Tiger mosquito (*Aedes albopictus*) development and immature survival are robust to artificial light at night, yet development varied more within certain light type and intensity combinations.

## INTRODUCTION

For millennia, life on Earth has experienced predictable light–dark cycles, and arthropods, like most organisms, have evolved to use these reliable cycles to time biological processes such as development, foraging and reproduction (Bradshaw and Holzapfel, 2007). Today, however, very few places remain untouched by artificial light at night (ALAN) (Falchi et al., 2016). Given its practical use, artificial light has been overlooked as an urban stressor, but  light pollution is now recognized as a pervasive driver of ecological change and a likely contributor to global arthropod declines (Bará and Falchi, 2023; Boyes et al., 2021a; Davies and Smyth, 2018; Desouhant et al., 2019; Dominoni et al., 2016; Falchi et al., 2016; Owens et al., 2020). Light pollution can alter the life history and behavior of arthropods, including their movement patterns, foraging behavior, mate finding, mate choice, development time and physiological processes (Desouhant et al., 2019). In this study, we explored how light pollution affects arthropod development, which is unique primarily because it involves metamorphosis, where a single individual develops into new forms throughout its lifecycle, which can function and respond differently to environmental stressors (e.g. temperature) (Orlinick et al., 2024).

Two main mechanisms have been proposed to explain how ALAN might influence arthropod development: disruption of hormonal regulation and alteration of behavior. First, ALAN can have cascading effects on hormone regulation by disrupting the production of melatonin, which is synthesized and released in darkness and suppressed by light in many species (Bembenek et al., 2005; Hardeland and Poeggeler, 2003; Subala and Shivakumar, 2018). Melatonin stimulates the release of prothoracicotropic hormone (PTTH), which in turn promotes the biosynthesis of ecdysteroids that regulate molting (Gäde et al., 1997; Richter et al., 2000). Therefore, if ALAN suppresses melatonin production and consequently delays PTTH release or ecdysteroid synthesis, exposure during development may slow molting and lengthen the time required to reach adulthood. Second, ALAN may indirectly affect juvenile growth by altering behavior (Roberts, 2012). For instance, in mosquitoes, larvae of species that are diurnal as adults were found to move faster under darkness than in light, and in choice assays they consistently preferred the darkest habitats (Hug et al., 2025; Liu et al., 2022). Therefore, if ALAN reduces movement, including that required for foraging, juvenile development may slow. Together, both the hormone regulation and behavior alteration mechanisms predict that ALAN exposure will lengthen juvenile arthropod development.

However, empirical studies do not consistently support the predictions of slower or prolonged development, instead revealing a wide range of outcomes. In juvenile crickets, development – which takes several months – can be up to 10 days longer under ALAN, resulting in adults that are larger and more likely to reach adulthood (Durrant et al., 2018; Rebar et al., 2022). Furthermore, in larvae of the Central and South American mosquito *Anopheles darlingi*, development – which takes only 1–2 weeks – was 1 day longer under constant light (Santos et al., 2023). In contrast, ALAN exposure accelerated orb-weaver spider (*Eriophora biapicata*) development – which takes nearly a year – by about 1 month in males and 2 months in females, resulting in fewer molts and a smaller overall body size (Willmott et al., 2018). Likewise, in tiger moths *Arctia caja*, larval development – which takes around 200 days under control conditions – was reduced to an average of around 100 days under ALAN, which also resulted in higher mortality rates (Van de Schoot et al., 2025b). Yet, in a common garden study with two other moth species (*Ochropleura plecta* and *Agrotis exclamationis*) from different ALAN locations (high versus low), only one species (*A. exclamationis*) showed ALAN effects: first, moths originating from high ALAN locations developed faster than moths originating from low ALAN locations; second, regardless of origin, moths reared under dynamic ALAN – where lights turned on for 1 min pulses at variable times to mimic motion detection lights – developed about 4 days faster than those under continuous ALAN or control conditions (Van de Schoot et al., 2025a). Finally in fruit flies (*Drosophila melanogaster*), rearing under ALAN did not affect juvenile development time – which takes about 9 days – or the probability of eclosion (McLay et al., 2017). Importantly, these inconsistent results highlight the necessity of studying species-specific responses to ALAN because not only are there existing differences in development times among species – from days to weeks to months – but sometimes even closely related species, like the moths, develop at different rates and respond to ALAN in distinct ways.

Furthermore, most studies of ALAN impacts on arthropod development focus on simple presence/absence contrasts (light versus dark) (Durrant et al., 2018; Hoffman and Subramanian, 2005; McLay et al., 2017; Rebar et al., 2022; Santos et al., 2023; Van de Schoot et al., 2025a,b; Willmott et al., 2018). Yet urban environments differ not only in whether light is present but also in the intensity and spectral composition (Gaston et al., 2013; Hirt et al., 2023). This is especially relevant as many cities are transitioning from high-pressure sodium (HPS) lamps to broad-spectrum, blue-rich light-emitting diodes (LEDs), which are predicted to have stronger biological effects (Bará and Falchi, 2023; Gaston et al., 2013; Pawson and Bader, 2014). However, the developmental impacts of these realistic light regimes on arthropods remain largely understudied (Boyes et al., 2021b).

Understanding how ALAN impacts mosquitoes is of particular interest because they are prevalent urban pests and major vectors of human pathogens (Coetzee et al., 2022, 2023). ALAN has already been shown to alter circadian gene expression, diapause and behavior in adult mosquitoes (Duffield, 2024; Fyie et al., 2021, 2024; Johnson et al., 2025; Rund et al., 2020; Sheppard et al., 2017; Westby and Medley, 2020; Wolkoff et al., 2023). However, the developmental effects of ALAN on mosquitoes remain largely unexplored, aside from two laboratory studies which tested differences between constant light and constant dark regimes only (Hoffman and Subramanian, 2005; Santos et al., 2023). Given the strong relationship between larval development and female body size, fecundity and population growth in mosquitoes (Armbruster and Hutchinson, 2002; Costanzo et al., 2018), even subtle changes in development times could have consequences for mosquito ecology and vectorial capacity.

Here, we investigated how HPS- and LED-derived ALAN at varying intensities influence the development of *Aedes albopictus*, a globally invasive mosquito and important vector of arboviruses (e.g. dengue and chikungunya) that thrives in urban and suburban environments (Paupy et al., 2009; Westby et al., 2021; Yin et al., 2019). We focused on development during the summer growing season, when populations are actively developing. We conducted a semi-natural field experiment to measure development time, emergence and mortality in larvae exposed to different light types and intensities. Based on the proposed mechanisms of the hormonal and behavioral hypotheses and prior work showing that ALAN alters circadian gene expression, diapause and other behaviors in adult *Ae. albopictus*, we predicted that ALAN would extend larval development time with stronger effects under higher intensities and under LED compared with HPS lighting. By manipulating both the light spectrum and intensity, our study moves beyond presence/absence comparisons to investigate how the characteristics of ALAN may shape the life history of mosquitoes.

## MATERIALS AND METHODS

To assess how LED and HPS light pollution at different intensities affect the development of *Aedes albopictus* (Skuse 1894), during the summer of 2024 we conducted a semi-natural field experiment under the canopy of the oak-hickory forest at Tyson Research Center. Tyson Research Center is a 2000 acre, primarily forested, environmental field station bordered by state and county parks (38°31′N, 90°33′W) located approximately 38 km outside St Louis, MO, USA. No detectable artificial illumination or sky glow was present at the site of the experiment. The experiment was designed in a dose–response fashion to identify biologically significant spectra and intensity of light pollution.

**Fig. 1.**
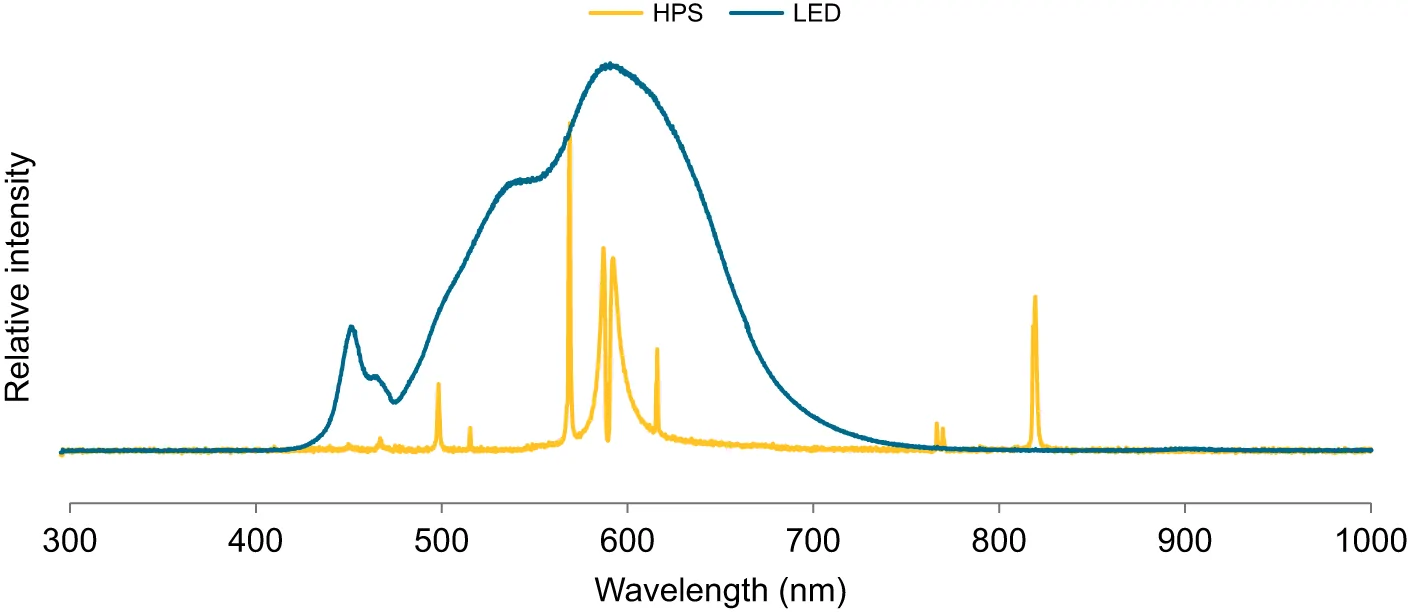
**Relative spectral emission of the light-emitting diode (LED) and high-pressure sodium (HPS) lamps used in the light treatments.** The LED light produced a broad, continuous emission profile across the visible spectrum, including shorter blue wavelengths that are often more biologically sensitive in many organisms, whereas the HPS light produced distinct emission peaks in the yellow–orange region.

We set up three light type treatments: HPS, LED and control. Portable power stations with solar chargers powered both HPS and LED lamps. The HPS lamp (35 W, Eaton Lighting, W-35-H/PC) was secured to a tree, and the LED lamp (400 lumens, 3250 K, Goal Zero Skylight Portable Area Light) was secured to the ground. The HPS lamp was programmed to turn on at dusk and off at dawn, whereas the LED lamp had to remain on continuously because of unalterable electrical specifications that prevented us from using outlet timers (the light would automatically switch to running on internal battery when an outlet timer shut off electrical current from the generator). Emission spectra were measured with an integration time of 100 ms using a USB4000 spectrometer (Ocean Optics) equipped with VIS-NIR fiber patch cord as a probe and controlled via SpectraSuite software (Ocean Optics) (Fig. 1). Each treatment table (2.4×0.9 m) and lamp were set up at appropriate distances (14 m HPS to LED; 32 m HPS to control; 20 m control to LED) so that the lamps from the LED and HPS treatments did not illuminate other treatment tables. The HPS and control tables were positioned beneath a continuous forest canopy and remained shaded from direct sunlight. Because of constraints associated with the power station and solar panel charging set-up, the LED table was also positioned under continuous canopy but was within a few meters of a canopy opening. Because low-angle sunlight could partially reach the end of this table nearest the opening in the late afternoon, we secured two temperature loggers (Lascar EL-USB-1-LCD) on the LED table: one at the end nearest the canopy opening and one at the end farther beneath the canopy. We also placed one temperature logger each on the HPS and control table. All loggers recorded ambient temperature hourly throughout the experiment, allowing us to assess potential microclimatic differences among treatments and across the LED table.

**Fig. 2.**
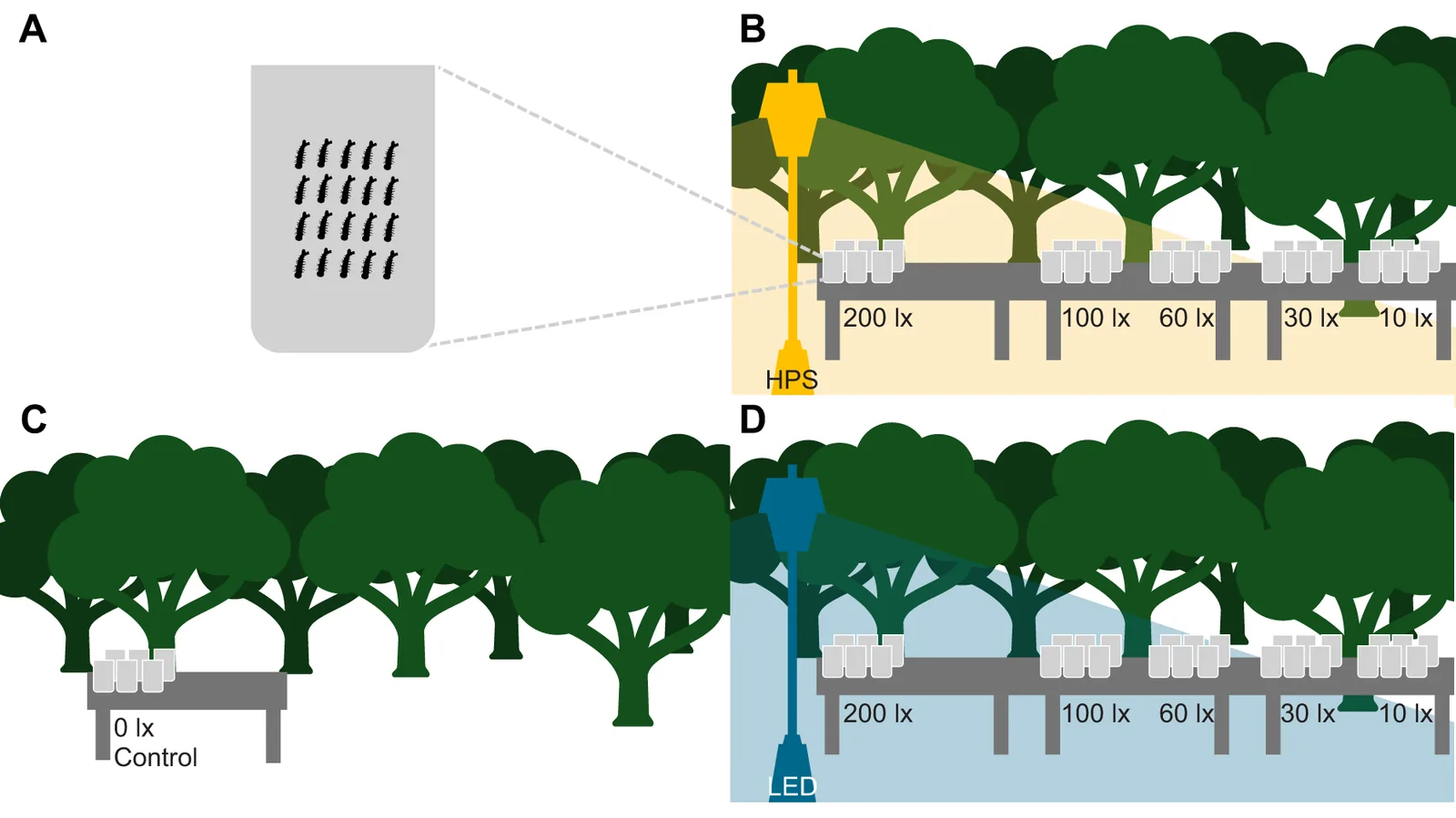
**Diagram of experiment design.** (A) Depiction of the 20 larvae within each of the six replicate cups per light type and intensity treatment. (B) Depiction of the HPS treatment group with different intensity treatments (10/30/60/100/200 lx). (C) Depiction of the control treatment group (no artificial light exposure; natural dark conditions). (D) Depiction of the LED treatment group with different intensity treatments (10/30/60/100/200 lx).

Mosquito larvae were placed in 0.4 l translucent plastic cups on the treatment tables at distances from the lamps corresponding to the gradient of intensity treatments of 10, 30, 60, 100 and 200 lx (i.e. 10 lx was furthest from the lamps; Fig. 2), which were based on levels used in laboratory experiments on *Ae. albopictus* adults (Kawada et al., 2005). Illuminance (lx) was measured using an MT-912 light meter (Shenzhen Flus Technology Co., Ltd). Each light type and intensity combination consisted of six replicate cups, each containing 20 mosquito larvae (*n*=120 for each light type and intensity combination). The larvae were hatched from F_5_ eggs collected from a laboratory colony that originated from urban St Louis, MO, USA. The lab colony was maintained with standard mosquito-rearing protocols: all life stages were maintained in environmental chambers at 25°C and a 16 h:8 h light:dark cycle. Eggs were stored in transparent plastic boxes with 90–100% relative humidity (RH). Each generation of larvae was reared in a single pan with 3 l of water, 0.6 g bovine liver powder and 400 larvae. Adults were given *ad libitum* access to a 10% sugar solution and fed on defibrinated sheep blood through a hog intestine membrane. For this experiment, F_5_ eggs were hatched in deionized water in 0.4 l translucent plastic beakers with 0.3 g l^−1^ of nutrient broth on 18 July 2024. Approximately 24 h after hatching, the first instar larvae were randomly assigned and deployed to one of 6 replicates within each light type and intensity combination (Fig. 2).

Each replicate contained 20 mosquito larvae and 330 ml of deionized water. Translucent cups had a water overflow drilled on the side of the container near the top that was covered with white mosquito netting to prevent larvae from being washed out. The tops of the cups were also covered with white netting and secured with rubber bands to prevent oviposition by wild mosquitoes. The translucent cups were placed in transparent plastic trays, with holes drilled in the bottom to prevent water from accumulating, that were secured with zip ties to the appropriate treatment table. Larvae in each cup were fed with 0.03 g of bovine liver powder (0.0015 g per larva) at the beginning of the experiment. The cups were monitored daily for the presence of pupae. The first pupae were detected and plucked on 25 July 2024 and transferred to separate transparent conical vials covered with white mosquito netting; the vials were placed in translucent cups held in transparent plastic trays that were secured adjacent to the larval pans in the field. We also added an additional 0.015 g of liver powder on 26 July 2024 to all cups containing larvae. The date of adult emergence and sex were recorded for each individual in each replicate. The number of individuals that did not survive to adult emergence was also recorded. Within each light type and intensity group, replicates were rotated every couple of days to ensure that minor differences in position to the light source would not impact the experiment. The experiment concluded on 21 August 2024.

### Microclimate analysis

To assess temperature variation, we first calculated the minimum, maximum, mean and standard deviation of temperature throughout the experiment at each of the three treatment tables (control, HPS and LED). We then tested whether hourly ambient temperature differed among treatment tables using a one-way ANOVA with treatment table as the explanatory variable. When the overall treatment effect was significant, we used a *post hoc* Tukey test to assess significant pairwise contrasts across groups.

### Development analysis

Our overarching hypothesis is that ALAN will alter mosquito development by disrupting circadian and hormonal pathways that regulate life-history timing. To assess the effect of light type and intensity of ALAN on mosquito time to pupation and emergence, we fitted generalized linear mixed models (GLMM) using the ‘glmmTMB’ package in R (Brooks et al., 2017; McGillycuddy et al., 2025) (https://www.r-project.org/). Time to pupation and time to emergence were modeled separately, and because male and female mosquitoes are known to differ in development time, emergence models were fitted separately by sex. Throughout, ‘replicate’ refers to an individual cup containing 20 larvae (*n*=6 cups per light type and intensity combination); replicate was included as a random effect in all models to account for non-independence among larvae within the same cup. Preliminary checks indicated underdispersion in time to pupation and emergence, so we modeled time to pupation and emergence using a Conway–Maxwell Poisson distribution (Sellers et al., 2012).

We fitted three complementary models to ask three questions: (1) does ALAN affect development relative to ambient darkness; (2) does development respond to intensity within illuminated treatments; and (3) does development differ among specific light type/intensity combinations relative to ambient darkness?

First, we tested the prediction that exposure to ALAN, regardless of intensity, alters development relative to natural dark conditions. We fitted a GLMM with light type (control, HPS, LED) as a fixed effect and replicate as a random effect. This model tested for overall differences among light environments without partitioning variation by intensity, serving as the simplest test of whether the presence of light pollution at night affects development relative to darkness.

Second, we tested the prediction that mosquito development responds to light intensity within illuminated treatments. Because the control group has no comparable intensity value, it was excluded from this analysis. We fitted a GLMM with light type (HPS or LED) as a categorical fixed effect, intensity (10, 30, 60, 100, 200 lx) as a continuous fixed effect, their interaction as a fixed effect, and replicate as a random effect. This model evaluated whether development time changed as a function of increasing light intensity and whether this relationship differed between light types.

Third, we tested the prediction that development differs among specific light type/intensity combinations relative to natural dark conditions (control). Because the control group was not run as a set of independent 0 lx replicates crossed with each light type, it could not be incorporated as an intensity level within a fully crossed light type by intensity factorial without duplicating replicates across light type. We therefore fitted a GLMM with a single 11-level light type/intensity treatment factor (control, HPS at 10/30/60/100/200 lx and LED at 10/30/60/100/200 lx) as a fixed effect and replicate as a random effect, allowing us to capture differences among specific light type/intensity combinations and the control.

In addition to mean time to pupation and emergence, we tested the prediction that ALAN influences phenotypic variability in development timing. Previous work has demonstrated that while urbanization can decrease or increase mean trait values or even leave them unchanged, it often leads to an increase in trait variability (Thompson et al., 2022). To visualize development variability, we calculated the coefficient of variation (CV) – an independent measure of variability that discounts the standard deviation by the mean – for time to pupation and emergence within each light type and intensity combination (Gotelli and Ellison, 2018). To formally test for differences in variability independent of any shift in mean development time, we modeled the dispersion parameter of each of the three models above as a function of the same fixed effect structure used in its conditional model. We compared each dispersion model with its corresponding constant dispersion model using a likelihood ratio test.

### Mortality analysis

To assess whether ALAN affected survival, we tested the prediction that mortality differs among light type and intensity treatments. We fitted a GLMM with a binomial distribution, with survival as the response variable (1=survived to emergence, 0=did not survive to emergence), replicate as a random effect, and light type/intensity treatment group as a fixed effect.

## RESULTS

### Temperature

**Fig. 3.**
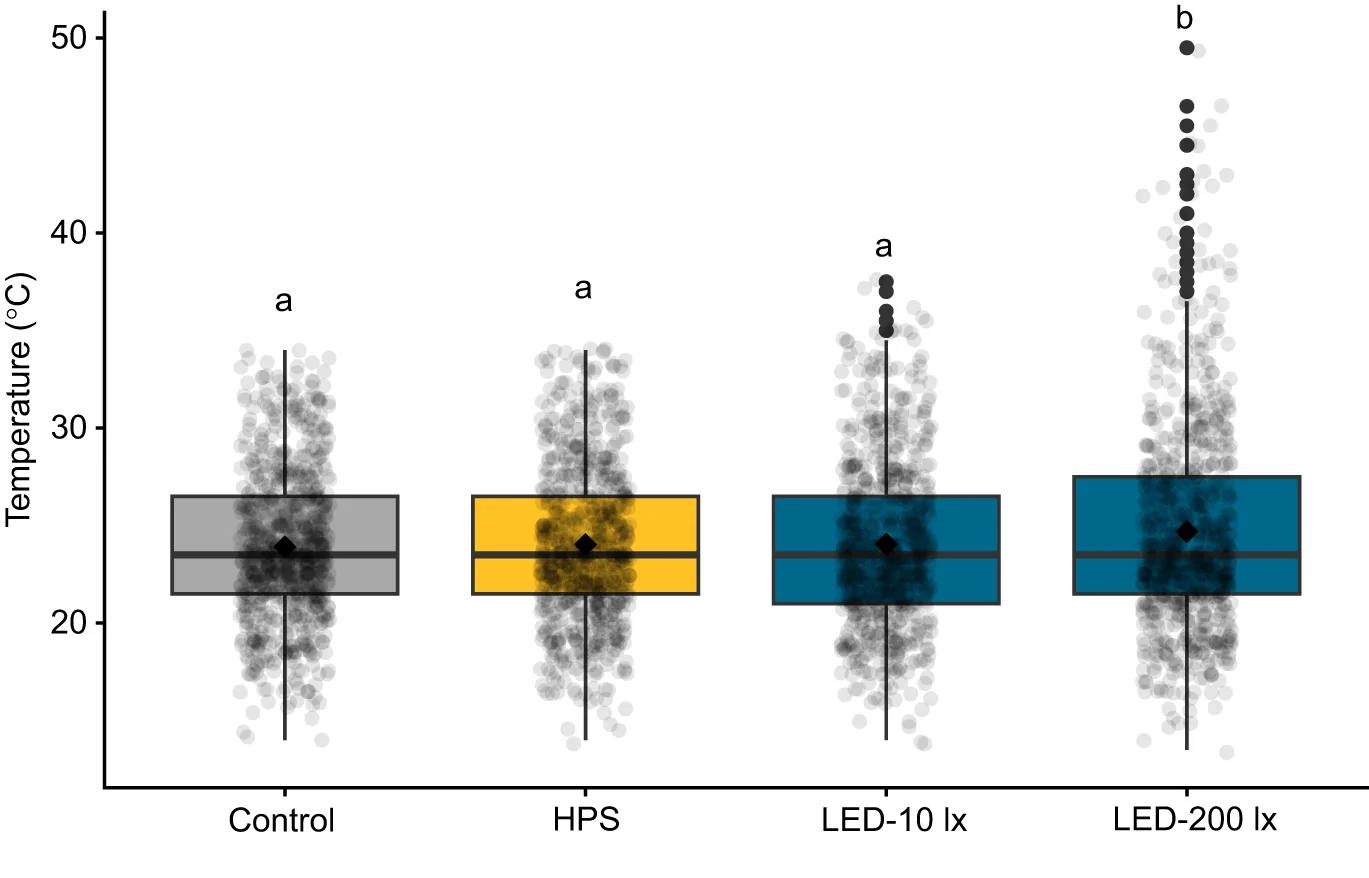
**Variation in temperature at the different experimental tables for each treatment.** Temperature readings were taken every hour throughout the entirety of the experiment. Given the variation in canopy cover over the LED treatment table, we had a logger at both ends of this treatment table (LED-200 lx and LED-10 lx, corresponding to the end nearest to and furthest from the lamps, respectively). Box plots show median, upper and lower quartiles and 1.5× the interquartile range; black circles are outliers; gray circles are individual data points, and black diamonds represent the mean. Different letters indicate significant differences between groups as determined by a one-way ANOVA followed by *post hoc* Tukey test (*P*<0.05).

The range in temperature experienced at the control and HPS treatment tables was nearly identical (Table 1, Fig. 3). Although the 10 lx side of the LED treatment table (LED-10 lx) experienced a 3.5°C higher maximum temperature than the control and HPS treatment tables, we did not detect any statistical differences between the groups (Table 1, Fig. 3). However, the 200 lx side of the LED treatment table (LED-200 lx) experienced a higher maximum temperature than the other three groups, with a maximum temperature that was 12°C higher than the LED-10 lx side of the table (Table 1, Fig. 3). This resulted in the temperature at the LED-200 lx side of the table being statistically higher on average than that of the other treatment tables by 0.65°C to 0.79°C (*F*_3,3229_=5.22, *P*=0.0014).

**Table 1.** Summary of temperature variation across treatments

Treatment	Temperature (°C)			
	Minimum	Maximum	Range	Mean±s.d.
Control	14.0	34.0	20.0	23.91±3.92
HPS	14.0	34.0	20.0	24.03±4.08
LED-10 lx	14.0	37.5	23.5	24.05±4.27
LED-200 lx	13.5	49.5	36	24.69±5.33

HPS, high-pressure sodium; LED, light-emitting diode. For the LED treatment, a logger was placed at each end of the treatment table (corresponding to an intensity of 10 lx and 200 lx).

### Pupation

**Fig. 4.**
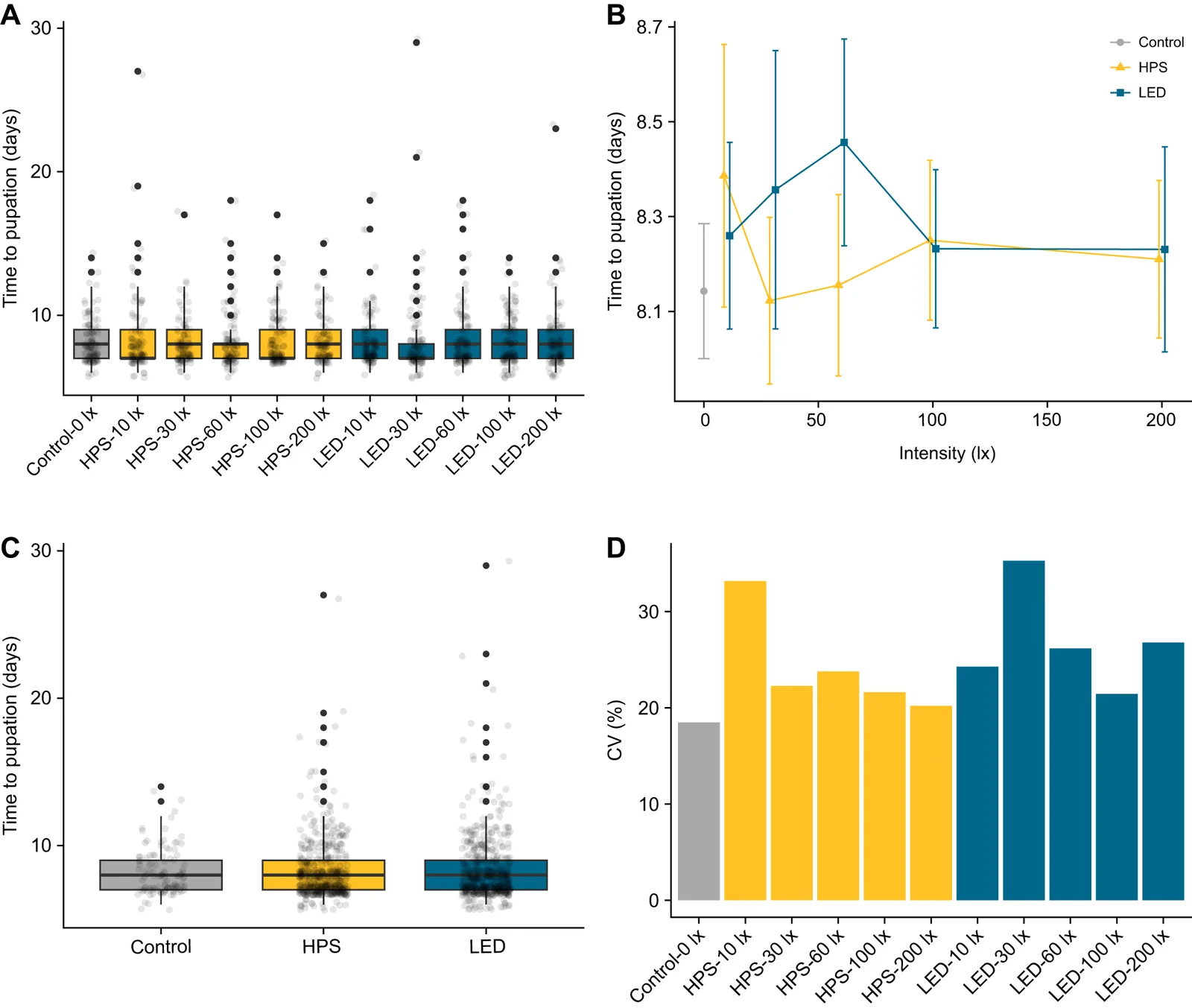
**Exposure to different types and intensities of artificial light at night (ALAN) did not cause significant differences in average time to pupation, but increased variation in development.** (A) Box plot of time to pupation, grouped by light type and intensity. (B) Line plot showing mean±s.e.m. time to pupation against light intensity for each light type. (C) Box plot of time to pupation, grouped by light type. (D) Bar plot highlighting the coefficient of variation (CV) within each light type and intensity combination.

Overall, the onset of pupation occurred between 6 and 29 days (*n*=1158 pupae, median=8 days, mean±s.d.=8.25±2.08 days, CV=25.24%). It is not feasible to determine the sex of larvae or pupae, so the sexes were not separated in the time to pupation analysis as they were in the time to emergence analysis (see below). There was minimal variation in the mean and median number of days to pupation across light types, intensities and replicates (Table 2; Table S1). Time to pupation showed no significant intensity-specific effects of light pollution (Table S2; Fig. 4A,B), nor were there differences among HPS, LED and control treatments when intensity was ignored (Table S3; Fig. 4C). Similarly, our analyses did not uncover any specific effects of light type or intensity when examining specific light type and intensity combinations (Table S4; Fig. 4A,B). However, variation in time to pupation became more evident when examining differences in the CV among treatment groups (Table 2, Fig. 4D). Notably, the control group had the lowest CV, while the greatest CV values were observed in the HPS-10 lx and LED-30 lx groups. Consistent with these patterns, likelihood ratio tests comparing each dispersion model to its corresponding constant-dispersion model indicated that variability in the onset of pupation differed significantly by light type (χ^2^_2_=18.477, *P*=9.723×10^−5^; Table S3), by light type and intensity among illuminated treatments (χ^2^_3_=18.757, *P*=3.069×10^−4^; Table S2), and among specific light type/intensity combinations (χ^2^_10_=61.318, *P*=2.039×10^−9^; Table S4), despite the absence of corresponding effects on mean time to pupation.

**Table 2.** Summary table of time to pupation, grouped by treatment

Light treatment	Intensity (lx)	*n*	Time to pupation (days)		CV (%)
			Median	Mean±s.d.	
Control	0	112	8	8.14±1.50	18.49
HPS	10	101	7	8.39±2.78	33.17
HPS	30	106	8	8.12±1.81	22.26
HPS	60	103	8	8.16±1.94	23.78
HPS	100	112	7	8.25±1.78	21.62
HPS	200	100	8	8.21±1.66	20.21
LED	10	104	8	8.26±2.00	24.27
LED	30	101	7	8.36±2.95	35.28
LED	60	103	8	8.46±2.21	26.17
LED	100	112	8	8.23±1.76	21.44
LED	200	104	8	8.23±2.20	26.78

### Adult emergence

**Fig. 5.**
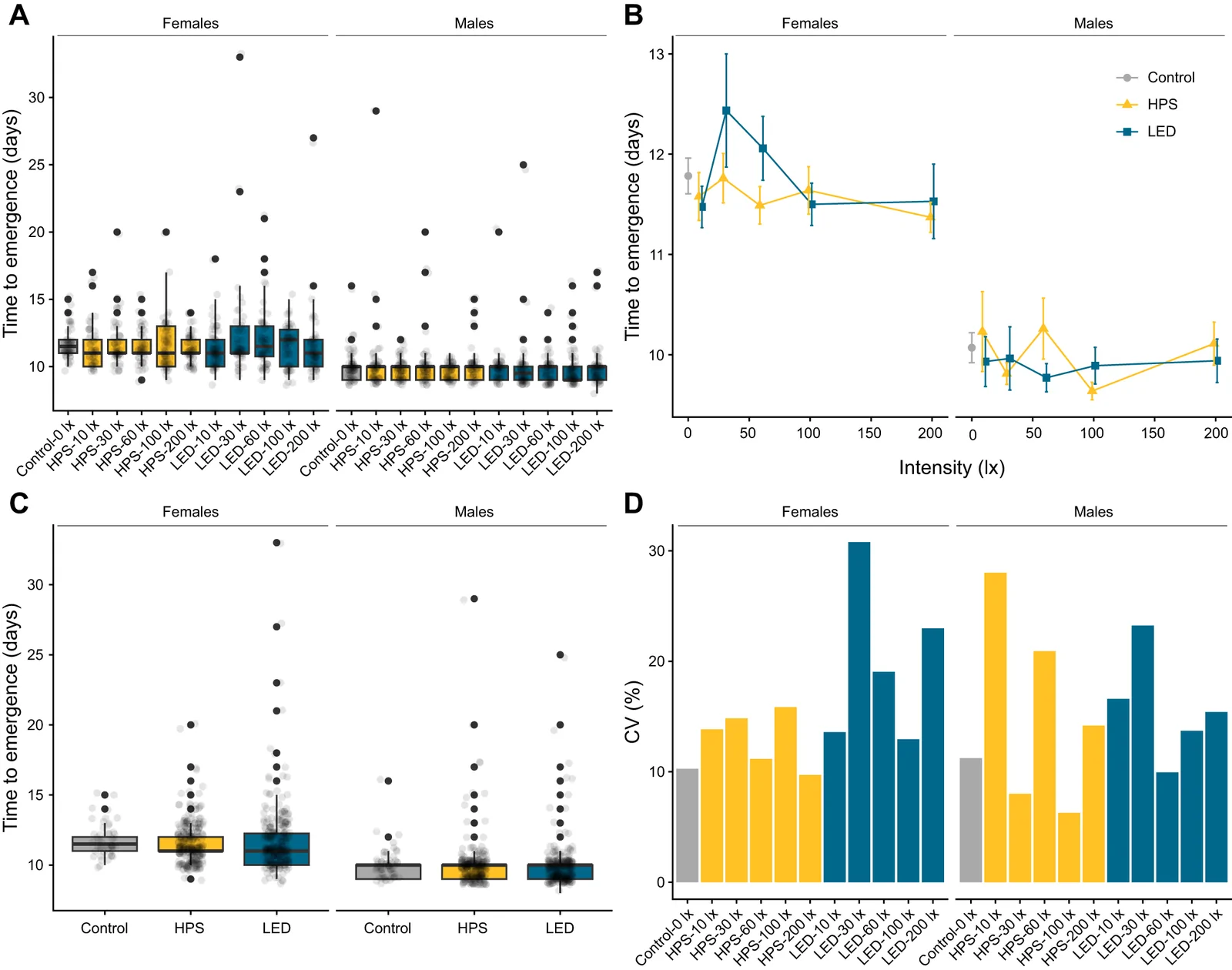
**Exposure to different types and intensities of ALAN did not cause significant differences in average time to adult emergence, but increased variation in development.** (A) Box plot of time to emergence, grouped by light type and intensity. (B) Line plot showing mean±s.e.m. time to emergence against light intensity for each light type. (C) Box plot of time to emergence, grouped by light type. (D) Bar plot highlighting the coefficient of variation (CV) within each light type and intensity combination. Data for females and males are shown separately in each panel (left and right, respectively).

Time to emergence naturally differs between male and female mosquitoes, with males emerging sooner than females (Bellini et al., 2018). Therefore, we analyzed the sexes separately. Overall, males (*n*=555 adults) in this experiment emerged between 8 and 29 days (median=10 days, mean±s.d.=9.97±1.66 days, CV=16.69%), while females (*n*=559 adults) emerged between 9 and 33 days (median=11 days, mean±s.d.=11.68±2.00 days, CV=17.16%). Similar to the time to pupation results, there was minimal variation in the average and median time to emergence within each sex across light types, intensities and replicates (Table 3; Table S5). Neither intensity-specific effects of light pollution (Tables S6 and S7; Fig. 5A,B), nor differences between HPS, LED and control treatments regardless of intensity (Tables S8 and S9; Fig. 5C), nor specific light type and intensity combinations (Tables S10 and S11; Fig. 5A,B) showed significant differences for either sex.

**Table 3.** Summary table of time to emergence, grouped by sex and treatment

Sex	Light treatment	Intensity (lx)	*n*	Time to emergence (days)		CV (%)
				Median	Mean±s.d.	
F	Control	0	46	11.5	11.78±1.21	10.26
F	HPS	10	45	11.0	11.58±1.60	13.84
F	HPS	30	50	11.0	11.76±1.74	14.83
F	HPS	60	47	11.0	11.49±1.28	11.17
F	HPS	100	61	11.0	11.64±1.84	15.84
F	HPS	200	54	11.0	11.37±1.10	9.71
F	LED	10	57	11.0	11.47±1.56	13.59
F	LED	30	46	11.0	12.43±3.83	30.78
F	LED	60	52	11.5	12.06±2.30	19.04
F	LED	100	50	12.0	11.50±1.49	12.94
F	LED	200	51	11.0	11.53±2.65	22.97
M	Control	0	57	10.0	10.07±1.13	11.24
M	HPS	10	52	10.0	10.23±2.87	28.02
M	HPS	30	53	10.0	9.81±0.78	8.01
M	HPS	60	50	10.0	10.26±2.14	20.91
M	HPS	100	47	10.0	9.64±0.60	6.28
M	HPS	200	45	10.0	10.11±1.43	14.18
M	LED	10	44	10.0	9.93±1.65	16.59
M	LED	30	54	9.5	9.96±2.31	23.23
M	LED	60	48	10.0	9.77±0.97	9.96
M	LED	100	55	9.0	9.89±1.36	13.71
M	LED	200	50	10.0	9.94±1.53	15.40

However, variation was more evident when examining the CV among treatments (Table 3, Fig. 5D). For females, the highest CV values were observed in the LED-30 lx and LED-200 lx groups. In males, LED-30 lx also had an elevated CV, but the greatest value was observed in the HPS-10 lx group. Controls consistently ranked among the lowest CV values for both males and females, and notably, no clear pattern emerged between CV and light intensity in either sex. As with pupation, likelihood ratio tests indicated that dispersion models significantly outperformed constant-dispersion models across all three treatment structures for both sexes, despite the lack of corresponding mean-level effects. In females, variability in emergence time differed by light type (χ^2^_2_=54.587, *P*=1.401×10^−12^; Table S8), by light type and intensity among illuminated treatments (χ^2^_3_=44.843, *P*=9.992×10^−10^; Table S6), and among specific light type/intensity combinations (χ^2^_10_=111.05, *P*<2.2×10^−16^; Table S10). In males, variability similarly differed by light type (χ^2^_2_=12.614, *P*=1.823×10^−3^; Table S9), by light type and intensity (χ^2^_3_=16.922, *P*=7.334×10^−4^; Table S7), and among specific light type/intensity combinations (χ^2^_10_=153.58, *P*<2.2×10^−16^; Table S11).

### Mortality

**Fig. 6.**
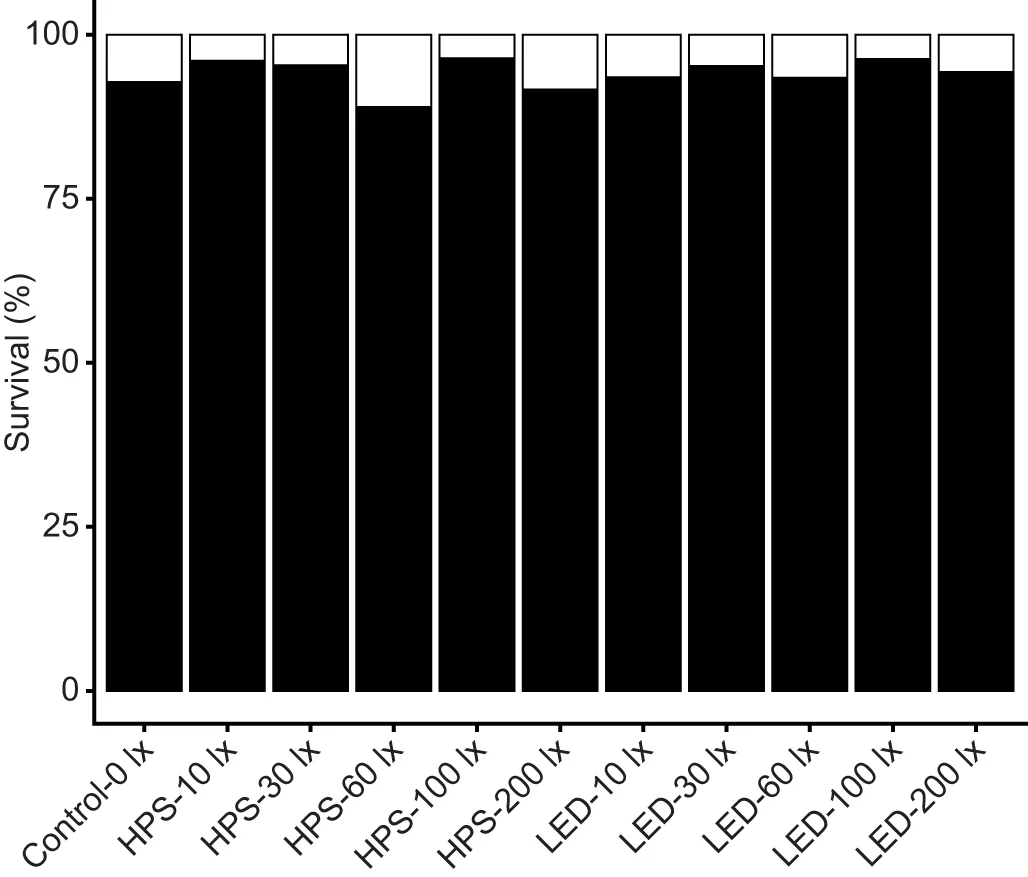
**Exposure to different types and intensities of ALAN did not affect the percentage of mosquito larvae that survived to adulthood.** Black bars indicate survival; white bars indicate the percentage of mosquito larvae that died.

Mortality across treatments was low overall (range: 3.57% to 11.01%; Fig. 6). The logistic regression did not identify significant differences in mortality across treatment groups (Table S12).

## DISCUSSION

Our study examined the impact of HPS and LED light pollution at varying intensities on the development of *Ae. albopictus* larvae. Contrary to our predictions, neither time to pupation nor emergence differed significantly across treatments, and mortality remained low. These results suggest that *Ae. albopictus* larval development is relatively resistant to ALAN under semi-natural field conditions. This pattern aligns with findings in fruit flies and some moth species (McLay et al., 2017; Van de Schoot et al., 2025a), where larval development was similarly unaffected, but contrasts with those from other arthropods, where development under ALAN was accelerated (e.g. some moth species and spiders) or delayed (e.g. crickets and some mosquito species) (Durrant et al., 2018; Rebar et al., 2022; Santos et al., 2023; Van de Schoot et al., 2025a,b). It also differs from findings in adult *Ae. albopictus*, where consistent effects of ALAN on behavior and physiology have been documented (Fyie et al., 2024; Johnson et al., 2025; Kawada et al., 2005; Westby and Medley, 2020). Combined, these findings highlight that ALAN sensitivity can vary not only among taxa but also across life stages within the same species.

### Detecting ALAN effects depends on existing variation in arthropod development

ALAN has demonstrated varying effects on the development of arthropods, including both slower and faster development across species. These species exhibit a considerable range in their standard development times, with some, e.g. mosquitoes, developing in as little as days while others, such as crickets, moths and spiders, taking weeks to months to develop. Therefore, a 9.5% increase in development time under ALAN for *Gryllus veletis* field crickets corresponds to nearly 13 days (Rebar et al., 2022), while a 20% increase under constant light in *An. darlingi* mosquitoes translates to only 1.5 days (Santos et al., 2023). Conversely, a 19% reduction in development time under ALAN for female orb-weaver spiders (*Eriophora biapicata*) corresponds to nearly 63 days (Willmott et al., 2018). In our experiment, the mean time to pupation and time to emergence for males and females differed from the control treatment by only 0.58–2.00% across comparisons with the pooled HPS and LED treatments. These small relative differences are consistent with the absence of significant treatment effects. Together, these comparisons demonstrate the value of reporting relative effect sizes alongside absolute changes in development time, particularly when comparing ALAN responses among species with substantially different development periods.

Given the rapid development of mosquitoes, detecting small relative effects of ALAN may necessitate examining smaller temporal units than days. In the case of *An. darlingi*, even a subjectively short 1.5 day increase in development time represented a substantial 20% change (Santos et al., 2023), highlighting that relatively short absolute differences can represent substantial relative impacts in fast-developing species. In contrast, slower-developing species such as crickets, moths and spiders demonstrate more extended absolute changes for comparable percentage effects (Durrant et al., 2018; Rebar et al., 2022; Van de Schoot et al., 2025a, 2025b; Willmott et al., 2018). For rapidly developing mosquitoes, differences of only a few per cent may correspond to hours rather than days and may therefore be difficult to detect through daily observations. Future studies should consider monitoring developmental transitions at sub-daily intervals to increase their ability to detect these comparatively small temporal differences.

### Intensity and spectra of light pollution

The spectra and intensity of light pollution are important characteristics that could lead to divergent biological and ecological outcomes (Gaston et al., 2013; Hirt et al., 2023). As many cities are transitioning from HPS lamps to broad-spectrum, blue-rich LEDs, it is predicted that these new LEDs will have stronger biological effects because many arthropods are behaviorally and physiologically sensitive to blue, violet and near-ultraviolet wavelengths (Bará and Falchi, 2023; Deichmann et al., 2021; Donners et al., 2018; Gaston et al., 2013; Longcore et al., 2015, 2018; Pawson and Bader, 2014). For example, caterpillars from LED-illuminated hedgerows had significantly heavier biomasses than caterpillars from either unlit or HPS-illuminated hedgerows, and there were fewer caterpillars per LED-illuminated grass transect than either unlit or HPS-illuminated grass transects (Boyes et al., 2021b). Furthermore, both LED and HPS lights reduced diapause incidence in *Ae. albopictus*, but the magnitude of the effect differed by spectra. In one study, diapause was reduced from 99.6% in non-ALAN treatment groups to 21.8% in LED-illuminated treatment groups (Fyie et al., 2024), whereas in a different study, diapause incidence was reduced from 99% in non-ALAN treatment groups to only 54% in HPS-illuminated treatment groups (Westby and Medley, 2020). In contrast, we found no evidence that spectrum altered *Ae. albopictus* development, suggesting that spectral sensitivity may be species specific, life-stage specific or physiological-process specific.

We also found no evidence that intensity of light pollution is important for development. We selected a range of light intensities that are observed in local urban areas (10 to 200 lx) because we know that there can be species-specific threshold effects in adult mosquitoes, where once a certain intensity of light pollution is reached, there is an abrupt biological response (Johnson et al., 2025; Kawada et al., 2005). For example, nocturnal host seeking was activated in *Aedes aegypti* adults at light pollution levels as low as 0.1 lx, but it took levels greater than 10 lx to activate host seeking in *Ae. albopictus* (Kawada et al., 2005). We know from findings in *An. darlingi* that light impacts development; mosquitoes reared under constant light conditions took longer to develop than those reared under normal light–dark conditions (Santos et al., 2023). However, that study was not simulating light pollution levels but rather constant daylight conditions with high intensities of 3000 to 5000 lx (Santos et al., 2023). So, although there is evidence that light does influence development, the intensities created by light pollution might not be biologically meaningful enough to impact it, further supporting the conclusion that light pollution sensitivity may be not only species specific but also life stage specific.

### Could temperature mask the effects of light pollution on mosquito development?

It is well established that organisms have thermal ranges within which development and growth can occur, and the shape of the relationship – the thermal performance curve – is left skewed. Performance gradually increases as temperatures approach an optimum, and then declines steeply once high temperatures start to disrupt cellular processes (Abarca et al., 2024). For example, in mosquitoes, *Ae. aegypti* reared at a constant 25°C develop faster than those reared at a constant 19°C (Torres et al., 2022), and *Ae. albopictus* reared at a constant 26°C develop faster than those reared at a constant 22°C (Alto and Juliano, 2001). Yet, in both studies, development did not continue to accelerate at 30°C, suggesting an upper thermal limit beyond which increasing temperature no longer results in faster development (Alto and Juliano, 2001; Torres et al., 2022) (see also Monteiro et al., 2007). At sufficiently high temperatures, development can slow and mortality increase, as demonstrated in *Ae. aegypti*, where exposure to constant 39°C caused nearly complete mortality, and the few individuals that reached pupation did so at times similar to individuals reared at 20°C (Carrington et al., 2013).

Temperature also fluctuates over the course of a 24 h day, meaning that development measured under constant conditions may not reflect responses under natural diel regimes. For example, in *Anopheles stephensi*, both 8°C and 12°C diel fluctuations accelerated immature development around a low mean of 18°C, had no detectable effect around an intermediate mean of 26°C, and slowed development around a high mean of 32°C relative to constant-temperature treatments with the same respective means (Paaijmans et al., 2013). In *Ae. aegypti*, large 18.6°C diel fluctuations around an average 26°C rearing temperature increased development time relative to a constant 26°C (Carrington et al., 2013). Together, these studies demonstrate that both the direction and magnitude of the impact of temperature on development time depend on both the mean temperature and the extent of fluctuation around that mean.

Prior studies demonstrating effects of ALAN on arthropod development have largely been conducted in the lab under constant or simplified temperature regimes (Durrant et al., 2018; Rebar et al., 2022; Santos et al., 2023; Van de Schoot et al., 2025a,b; Willmott et al., 2018). We instead conducted our experiment under naturally fluctuating thermal conditions in the field. We did this in order to characterize the effects of ALAN under ecologically realist thermal conditions. Our goal was to maintain comparable temperatures among light treatments, and we largely achieved this, although the field layout prevented perfect thermal matching. The control and HPS loggers recorded identical minimum and maximum temperatures of 14°C and 34°C, and nearly identical means of 23.91 and 24.03°C, respectively. The LED-10 lx logger recorded a similar mean of 24.05°C, and its overall range was slightly broader at 14–37.5°C.

The exception was the LED-200 lx logger. It recorded a mean of 24.69°C, only 0.64–0.78°C higher than the other logger locations, but it recorded a substantially broader range of 13.5–49.5°C. These temperature spikes up to 49.5°C require cautious interpretation because the logger's sensor was enclosed within black housing and was not protected by a radiation shield. Direct solar radiation can heat both a sensor and its housing, positively biasing air temperature measurements, particularly under high radiation and low wind conditions (Maclean et al., 2021). The highest readings occurred when low-angle, late-afternoon sunlight could reach the exposed end of the LED table. Therefore, these readings may reflect radiative heating of the logger rather than equivalent increases in ambient air temperature. Nevertheless, direct sunlight could also have warmed the nearby rearing cups, so the probable measurement bias does not necessarily mean that thermal differences were entirely absent.

The logger measurements, however, cannot be assumed to represent the water temperatures experienced by the larvae. Larvae were immersed in water within translucent cups, which were separated by an air gap from the surrounding transparent larval pans. Because water has greater thermal inertia than the thin logger housing, the water cups would be expected to heat and cool more slowly, buffering short-lived radiative temperature spikes. The surrounding pans and air gap would have further altered heat transfer relative to the directly exposed black logger. Because we did not measure water temperature within the rearing cups, we cannot determine whether water temperature approached the maxima recorded by the logger or how long any increases in water temperature persisted.

We therefore cannot entirely rule out the possibility that temperature specifically influenced larvae in the high-intensity LED treatments differently from those in the low-intensity LED, HPS and control treatments. If the brief window of direct sunlight produced a meaningful enough increase in water temperature to accelerate development, and if high-intensity LED light pollution meaningfully slowed development in equal magnitude, we could get the same null result observed here. However, we believe that this is unlikely because the LED-200 lx logger was on average only 0.64–0.78°C warmer than the other loggers because of the limited number of extremely high temperature recordings. Additionally, prior laboratory research demonstrates that large diel fluctuations can reduce the accelerating effects of higher mean temperatures (Carrington et al., 2013; Paaijmans et al., 2013).

These results highlight the challenge of measuring consistent thermal conditions in field and semi-field experiments and the importance of distinguishing ambient air temperature from the operative temperature experienced by aquatic organisms. Prior research has demonstrated that ALAN and temperature can interact to shape biological responses (Dominoni et al., 2020; Miller et al., 2017). Future studies should therefore continue to test ALAN under realistic diel temperature fluctuations while directly measuring water temperature within replicate rearing cups. This approach would help determine whether natural thermal variation attenuates ALAN effects, while avoiding uncertainty caused by radiative measurement error.

### Variation and trait consistency

Although mean development times were unaffected, we observed substantial differences in variation across treatments, suggesting that ALAN may influence developmental consistency – an aspect of traits with ecological importance that can be overlooked when focusing solely on population averages (Thompson et al., 2022). In particular, several LED and HPS treatments showed substantially higher variability in time to pupation and emergence compared with controls, whereas others showed very low variability. These inconsistencies suggest that ALAN does not uniformly impact development, potentially increasing heterogeneity in some conditions while reducing it in others. This pattern aligns with findings that show that while urbanization can decrease or increase mean trait values or even leave them unchanged, trait variability often increases with urbanization (Thompson et al., 2022). Increased variability in development may reflect subtle stress responses or heterogeneous strategies within a population exposed to novel environmental conditions such as ALAN. Thus, while our study detected no average shifts in *Ae. albopictus* development under ALAN, the changes we observed in variation, particularly under LED treatments, point to potential ecological effects that may only be evident when trait consistency is explicitly considered. Replication with additional populations and settings will be important to confirm whether these shifts are general and repeatable or context dependent.

### Implications for vector ecology

Artificial light at night is increasingly recognized as an urban driver of mosquito ecology, with potential consequences for both nuisance biting and disease transmission (Coetzee et al., 2022, 2023). Prior work has shown that light pollution can be a stronger predictor of mosquito-borne West Nile virus risk than other urban metrics, such as human footprint or impervious surface cover (Kernbach et al., 2021). Light pollution can also change the time of day mosquitoes bite, potentially impacting vector–host interactions (Duffield, 2024; Johnson et al., 2025; Rund et al., 2020; Sheppard et al., 2017). Our study adds nuance to this discussion by showing that typical levels of ALAN did not alter *Ae. albopictus* larval development timing or survival, suggesting that light pollution may not directly accelerate or delay generation time in urban environments. However, larval development conditions are tightly linked to adult phenotypes that underlie vectorial capacity (Araújo et al., 2012; LaDeau et al., 2015; Moller-Jacobs et al., 2014; Shapiro et al., 2016). Development time and resource availability influence female body size, which in turn is associated with fecundity, and population growth, as well as traits more directly tied to transmission, including daily survival, longevity, biting rate and vector competence (Araújo et al., 2012; Armbruster and Hutchinson, 2002; Costanzo et al., 2018; Hawley, 1985; LaDeau et al., 2015; Moller-Jacobs et al., 2014; Shapiro et al., 2016). Therefore, the increased variation in development observed under some ALAN treatments may still influence adult phenotypes and, consequently, transmission dynamics, even in the absence of shifts in mean development time.

These conclusions should be interpreted with several limitations in mind. Our design was restricted to a single field season, a limited set of spectra and intensities, and semi-natural containers where predation and competition were excluded. Such conditions may underestimate the ecological effects of ALAN in more complex urban habitats. Future work should extend these experiments across multiple generations, incorporate predator–prey and competitive interactions, and examine other mosquito vectors and pathogens under a broader range of urban and natural environments. These next steps will be critical for clarifying whether ALAN influences mosquito ecology primarily through larval development, adult behavior or broader community-level processes. Ultimately, understanding these pathways will help refine predictions of how urban lighting contributes to vector-borne disease risk and guide more effective management strategies.

## Supplementary Material

10.1242/jexbio.252361_sup1 Supplementary information
