# Supplementary material for: Impact of light pollution spectra and intensity on the development and mortality of tiger mosquito (Aedes albopictus) larvae
Source: J Exp Biol. 2026 Sep 17;229(18):jeb252361. doi: 10.1242/jeb.252361 (PMC13615692; doi:10.1242/jeb.252361)
Supplement: Supplementary file 1 [file jexbio-229-252361-s1.pdf]

Table S1. Days to pupation summary table grouped by replicate and treatment.

Treatment	Light	Intensity	Rep	n	median	mean	sd	CV
Control-0	Control	0	1	19	8.0	8.421053	1.8353259	21.79449
Control-0	Control	0	2	19	8.0	8.157895	1.0678721	13.09005
Control-0	Control	0	3	18	7.5	8.166667	1.7904978	21.92446
Control-0	Control	0	4	18	7.5	8.277778	1.6735153	20.21696
Control-0	Control	0	5	20	8.0	8.000000	1.1697953	14.62244
Control-0	Control	0	6	18	7.0	7.833333	1.5048940	19.21141
HPS-10	HPS	10	1	19	8.0	9.789474	5.2975775	54.11504
HPS-10	HPS	10	2	17	8.0	7.647059	0.9314757	12.18084
HPS-10	HPS	10	3	17	7.0	8.058824	1.5600716	19.35855
HPS-10	HPS	10	4	17	7.0	8.235294	1.6781467	20.37750
HPS-10	HPS	10	5	18	7.0	8.166667	2.1760731	26.64579
HPS-10	HPS	10	6	13	8.0	8.230769	1.8327505	22.26706
HPS-30	HPS	30	1	18	8.0	7.777778	0.8782038	11.29119
HPS-30	HPS	30	2	17	8.0	8.529412	2.5767627	30.21032
HPS-30	HPS	30	3	17	7.0	7.647059	1.0571883	13.82477
HPS-30	HPS	30	4	21	8.0	8.380952	2.4181851	28.85334
HPS-30	HPS	30	5	15	8.0	8.466667	1.7265435	20.39225
HPS-30	HPS	30	6	18	7.5	7.944444	1.4337209	18.04684
HPS-60	HPS	60	1	20	8.0	8.200000	1.5761379	19.22119
HPS-60	HPS	60	2	18	8.0	8.000000	1.3284223	16.60528
HPS-60	HPS	60	3	19	8.0	8.894737	2.9039195	32.64762
HPS-60	HPS	60	4	18	7.5	8.277778	2.3213980	28.04373
HPS-60	HPS	60	5	12	7.0	7.333333	0.8876254	12.10398
HPS-60	HPS	60	6	16	7.0	7.875000	1.5000000	19.04762
HPS-100	HPS	100	1	20	7.5	8.250000	1.4464112	17.53226
HPS-100	HPS	100	2	18	7.0	8.555556	2.7700546	32.37726
HPS-100	HPS	100	3	19	8.0	8.684211	2.0290291	23.36458
HPS-100	HPS	100	4	18	8.0	8.111111	1.3234931	16.31704
HPS-100	HPS	100	5	18	7.0	8.000000	1.5718105	19.64763
HPS-100	HPS	100	6	19	7.0	7.894737	1.2425215	15.73861
HPS-200	HPS	200	1	14	8.0	8.500000	1.5063966	17.72231

Journal of Experimental Biology • Supplementary information

HPS-200	HPS	200	2	17	7.0	7.764706	1.2004901	15.46086
HPS-200	HPS	200	3	16	8.0	8.187500	1.4244882	17.39833
HPS-200	HPS	200	4	15	8.0	8.733333	2.4630604	28.20298
HPS-200	HPS	200	5	20	8.0	8.550000	1.9324105	22.60129
HPS-200	HPS	200	6	18	7.0	7.611111	0.9785276	12.85657
LED-10	LED	10	1	18	7.0	7.777778	2.1297764	27.38284
LED-10	LED	10	2	18	7.0	8.555556	2.8946649	33.83375
LED-10	LED	10	3	17	8.0	8.470588	2.2112679	26.10525
LED-10	LED	10	4	14	8.0	7.785714	1.3114039	16.84372
LED-10	LED	10	5	19	8.0	8.473684	1.6454794	19.41870
LED-10	LED	10	6	18	8.0	8.388889	1.4199788	16.92690
LED-30	LED	30	1	18	8.0	8.555556	1.3814835	16.14721
LED-30	LED	30	2	16	7.0	7.375000	0.8062258	10.93187
LED-30	LED	30	3	14	8.0	8.142857	1.4600918	17.93095
LED-30	LED	30	4	17	7.0	8.588235	3.5891995	41.79205
LED-30	LED	30	5	19	7.0	9.263158	5.3108077	57.33258
LED-30	LED	30	6	17	7.0	8.000000	1.7677670	22.09709
LED-60	LED	60	1	19	7.0	7.842105	1.3849652	17.66063
LED-60	LED	60	2	15	7.0	7.466667	0.9154754	12.26083
LED-60	LED	60	3	15	8.0	8.666667	2.3804761	27.46703
LED-60	LED	60	4	20	8.0	9.150000	3.2163235	35.15108
LED-60	LED	60	5	17	8.0	9.058824	2.3040884	25.43474
LED-60	LED	60	6	17	8.0	8.411765	1.8391174	21.86363
LED-100	LED	100	1	19	8.0	8.157895	1.7404711	21.33481
LED-100	LED	100	2	19	7.0	8.000000	1.5634719	19.54340
LED-100	LED	100	3	18	8.5	8.666667	1.9703687	22.73502
LED-100	LED	100	4	18	7.0	7.555556	1.5038078	19.90334
LED-100	LED	100	5	18	8.0	8.444444	1.7895850	21.19245
LED-100	LED	100	6	20	8.5	8.550000	1.9594575	22.91763
LED-200	LED	200	1	19	8.0	9.157895	3.9758040	43.41395
LED-200	LED	200	2	18	7.0	7.722222	1.3197841	17.09073
LED-200	LED	200	3	16	8.0	8.875000	1.9958290	22.48821
LED-200	LED	200	4	14	7.5	7.642857	0.9287827	12.15230
LED-200	LED	200	5	19	8.0	7.894737	1.2425215	15.73861
LED-200	LED	200	6	18	7.5	8.000000	1.7489493	21.86187

Journal of Experimental Biology • Supplementary information

Table S2. Days to pupation GLMM conditional and dispersion model estimates, including light type, intensity, and their interaction as fixed effects, with replicate as a random effect for the conditional model. Controls are excluded from this model (n = 1,046).

Variable	Estimate	Standard Error	z-value	p-value
**Conditional Model**				
Light Type	0.01301	0.02280	0.57	0.568
Intensity	-0.00002920	0.0001581	-0.18	0.853
Light Type x Intensity	-0.00003869	0.0002192	-0.18	0.860
**Dispersion Model**				
Light Type	0.0264380	0.1366702	0.193	0.846612
**Intensity**	**-0.0033806**	**0.0008994**	**-3.759**	**0.000171**
**Light Type x Intensity**	**0.0022069**	**0.0012833**	**1.720**	**0.085487**

Table S3. Days to pupation GLMM conditional and dispersion model estimates with light type as fixed effect with levels of HPS, LED, and Control, and replicate as a random effect for the conditional model (n = 1,158). Estimated coefficients represent estimated differences from the control group.

Variable	Estimate	Standard Error	z-value	p-value
**Conditional Model**				
HPS	0.009903	0.024171	0.41	0.682
LED	0.019800	0.024141	0.82	0.412
**Dispersion Model**				
**HPS**	**0.4901**	**0.1489**	**3.290**	**0.001**
**LED**	**0.6705**	**0.1491**	**4.497**	**6.9×10^{-6}**

Journal of Experimental Biology • Supplementary information

Table S4. Days to pupation GLMM conditional and dispersion model estimates with treatment (combining light type and intensity categories) as a fixed effect and replicate as a random effect for the conditional model (n = 1,158). Estimated coefficients represent estimated differences from the control group.

Variable	Estimate	Standard Error	z-value	p-value
**Conditional Model**				
HPS-10	0.029086	0.031603	0.92	0.357
HPS-30	-0.002405	0.031477	-0.08	0.939
HPS-60	0.001493	0.031680	0.05	0.962
HPS-100	0.013014	0.030913	0.42	0.674
HPS-200	0.008438	0.031865	0.26	0.791
LED-10	0.014311	0.031488	0.45	0.649
LED-30	0.025887	0.031628	0.82	0.413
LED-60	0.037621	0.031372	1.20	0.230
LED-100	0.011083	0.030931	0.36	0.720
LED-200	0.010825	0.031518	0.34	0.731
**Dispersion Model**				
**HPS-10**	**1.0247**	**0.1981**	**5.173**	**2.30×10^{-7}**
HPS-30	0.2998	0.1944	1.542	0.123045
**HPS-60**	**0.4416**	**0.1957**	**2.257**	**0.024029**
HPS-100	0.2906	0.1912	1.520	0.128505
HPS-200	0.1660	0.1972	0.842	0.399701
**LED-10**	**0.4864**	**0.1955**	**2.489**	**0.012818**
**LED-30**	**1.1065**	**0.1984**	**5.576**	**2.46×10^{-8}**
**LED-60**	**0.6683**	**0.1961**	**3.408**	**0.000654**
**LED-100**	**0.3253**	**0.1914**	**1.700**	**0.089121**
**LED-200**	**0.6251**	**0.1957**	**3.194**	**0.001402**

Table S5. Days to emergence summary table grouped by replicate, sex, and treatment.

Sex	Treatment	Rep	n	median	mean	sd	CV
F	Control-0	1	10	12.0	11.7000	0.6749	5.769
F	Control-0	2	9	11.0	11.4444	0.7264	6.348
F	Control-0	3	6	12.5	12.3333	1.7512	14.199
F	Control-0	4	8	11.5	11.8750	1.6421	13.828
F	Control-0	5	6	11.5	11.6667	1.2111	10.3805
F	Control-0	6	7	11.0	11.8571	1.4638	12.346
F	HPS-10	1	6	11.0	11.1667	1.4720	13.182
F	HPS-10	2	6	11.0	11.0000	0.6324	5.750
F	HPS-10	3	8	12.0	11.6250	1.4079	12.111
F	HPS-10	4	10	11.5	11.7000	1.3375	11.432
F	HPS-10	5	6	12.0	12.3333	2.5820	20.935
F	HPS-10	6	9	11.0	11.5556	1.9436	16.8200
F	HPS-30	1	7	11.0	11.7143	1.6036	13.689
F	HPS-30	2	10	11.0	12.1000	3.1073	25.680
F	HPS-30	3	7	11.0	11.5714	1.3973	12.075
F	HPS-30	4	10	12.0	11.7000	0.8233	7.036
F	HPS-30	5	8	11.5	11.8750	1.3562	11.421
F	HPS-30	6	8	11.0	11.5000	1.4142	12.298
F	HPS-60	1	12	11.0	11.5833	1.5050	12.993
F	HPS-60	2	11	11.0	11.0909	1.4460	13.038
F	HPS-60	3	10	12.0	11.8000	1.0328	8.752
F	HPS-60	4	7	11.0	11.8571	1.2150	10.247
F	HPS-60	5	4	11.0	11.0000	0.8165	7.423
F	HPS-60	6	3	11.0	11.3333	1.5275	13.478
F	HPS-100	1	11	12.0	11.6364	1.2863	11.054
F	HPS-100	2	10	11.5	12.6000	3.3400	26.508
F	HPS-100	3	10	12.0	11.6000	1.5776	13.600
F	HPS-100	4	11	11.0	11.1818	1.6011	14.319
F	HPS-100	5	9	11.0	11.5556	1.3333	11.538
F	HPS-100	6	10	11.0	11.3000	1.1595	10.261
F	HPS-200	1	10	11.0	11.3000	0.8233	7.286

Journal of Experimental Biology • Supplementary information

F	HPS-200	2	7	11.0	10.7143	0.7559	7.055
F	HPS-200	3	10	11.0	11.4000	1.1738	10.296
F	HPS-200	4	9	11.0	11.2222	1.0929	9.739
F	HPS-200	5	10	12.0	12.2000	1.2293	10.076
F	HPS-200	6	8	11.0	11.1250	1.1260	10.121
F	LED-10	1	5	10.0	11.8000	3.4928	29.600
F	LED-10	2	9	10.0	11.0000	1.6583	15.076
F	LED-10	3	10	11.0	11.3000	0.8233	7.286
F	LED-10	4	8	11.0	11.2500	1.2817	11.393
F	LED-10	5	11	12.0	12.0000	1.4142	11.785
F	LED-10	6	14	11.0	11.5000	1.2860	11.1823
F	LED-30	1	13	11.0	12.6154	3.3301	26.397
F	LED-30	2	4	11.0	11.2500	1.2583	11.185
F	LED-30	3	8	11.0	11.5000	1.3093	11.385
F	LED-30	4	5	11.0	11.2000	0.8367	7.470
F	LED-30	5	7	13.0	15.8571	7.8407	49.446
F	LED-30	6	9	11.0	11.5556	1.8782	16.254
F	LED-60	1	6	11.5	11.3333	1.6330	14.409
F	LED-60	2	6	11.0	11.1667	0.9832	8.805
F	LED-60	3	9	12.0	11.7778	2.2791	19.351
F	LED-60	4	12	11.5	12.5000	3.1188	24.951
F	LED-60	5	9	12.0	12.7778	2.7739	21.709
F	LED-60	6	10	12.0	12.1000	1.6633	13.746
F	LED-100	1	8	12.0	11.5000	0.7559	6.573
F	LED-100	2	9	12.0	11.7778	1.2018	10.204
F	LED-100	3	9	12.0	11.4444	1.5899	13.892
F	LED-100	4	7	10.0	10.2857	1.3801	13.418
F	LED-100	5	8	11.0	11.5000	1.7728	15.416
F	LED-100	6	9	13.0	12.2222	1.7159	14.039
F	LED-200	1	6	11.0	13.6667	6.5625	48.018
F	LED-200	2	10	11.5	11.3000	1.3375	11.836
F	LED-200	3	7	12.0	13.0000	1.8257	14.044
F	LED-200	4	9	11.0	10.8889	0.6009	5.519

Journal of Experimental Biology • Supplementary information

F	LED-200	5	10	11.0	11.0000	1.2472	11.338
F	LED-200	6	9	10.0	10.4444	1.6667	15.957
M	Control-0	1	8	10.0	10.3750	2.3260	22.420
M	Control-0	2	9	10.0	10.1111	0.9280	9.178
M	Control-0	3	11	10.0	10.0909	0.7006	6.943
M	Control-0	4	9	10.0	10.2222	0.8333	8.152
M	Control-0	5	11	10.0	10.2727	0.9045	8.805
M	Control-0	6	9	9.0	9.3333	0.5000	5.357
M	HPS-10	1	11	10.0	12.0000	5.8992	49.160
M	HPS-10	2	10	10.0	9.8000	0.7888	8.049
M	HPS-10	3	8	10.0	10.0000	0.5345	5.345
M	HPS-10	4	7	9.0	9.2857	0.4880	5.255
M	HPS-10	5	12	10.0	9.9167	1.1645	11.743
M	HPS-10	6	4	9.5	9.5000	0.5774	6.077
M	HPS-30	1	12	10.0	10.0833	0.9003	8.929
M	HPS-30	2	6	9.5	9.5000	0.5477	5.766
M	HPS-30	3	10	10.0	9.8000	0.6324	6.454
M	HPS-30	4	8	9.0	9.2500	0.4629	5.004
M	HPS-30	5	7	10.0	10.0000	0.8165	8.165
M	HPS-30	6	10	10.0	10.0000	0.9428	9.428
M	HPS-60	1	8	10.0	9.8750	0.6409	6.490
M	HPS-60	2	7	10.0	10.1428	0.6901	6.803
M	HPS-60	3	9	9.0	11.2222	4.2065	37.483
M	HPS-60	4	11	10.0	10.3636	2.2923	22.118
M	HPS-60	5	8	9.5	9.5000	0.5345	5.626
M	HPS-60	6	7	10.0	10.2857	1.2536	12.187
M	HPS-100	1	9	9.0	9.3333	0.5000	5.357
M	HPS-100	2	8	9.5	9.5000	0.5345	5.626
M	HPS-100	3	6	10.0	9.8333	0.7528	7.655
M	HPS-100	4	7	10.0	10.0000	0.5774	5.774
M	HPS-100	5	8	10.0	9.6250	0.5175	5.377
M	HPS-100	6	9	10.0	9.6667	0.7071	7.315
M	HPS-200	1	4	10.0	10.7500	2.2174	20.626

Journal of Experimental Biology • Supplementary information

M	HPS-200	2	10	10.0	10.0000	1.1547	11.547
M	HPS-200	3	6	10.0	9.6667	0.5164	5.342
M	HPS-200	4	5	10.0	11.0000	2.2361	20.328
M	HPS-200	5	10	10.0	10.3000	1.8288	17.755
M	HPS-200	6	10	10.0	9.6000	0.5164	5.379
M	LED-10	1	13	10.0	9.7692	0.7250	7.421
M	LED-10	2	7	10.0	9.8571	0.3780	3.834
M	LED-10	3	7	10.0	11.1428	3.9340	35.305
M	LED-10	4	6	10.0	9.6667	0.5164	5.342
M	LED-10	5	7	10.0	9.5714	0.5345	5.584
M	LED-10	6	4	9.5	9.5000	0.5774	6.077
M	LED-30	1	5	9.0	9.6000	0.8944	9.317
M	LED-30	2	12	9.5	9.5000	0.5222	5.497
M	LED-30	3	6	10.0	10.0000	0.6324	6.324
M	LED-30	4	12	10.0	11.4167	4.6213	40.478
M	LED-30	5	12	9.0	9.2500	0.4523	4.889
M	LED-30	6	7	10.0	9.7143	0.7559	7.782
M	LED-60	1	13	9.0	9.4615	0.6602	6.978
M	LED-60	2	9	10.0	9.7778	0.6667	6.818
M	LED-60	3	5	11.0	10.6000	1.1402	10.756
M	LED-60	4	7	9.0	9.2857	0.7559	8.141
M	LED-60	5	7	10.0	10.4286	1.6183	15.518
M	LED-60	6	7	10.0	9.5714	0.5345	5.584
M	LED-100	1	11	10.0	10.274	2.0538	19.993
M	LED-100	2	9	9.0	9.3333	0.5000	5.357
M	LED-100	3	7	9.0	9.7143	1.4960	15.400
M	LED-100	4	11	10.0	10.1818	1.4709	14.447
M	LED-100	5	7	10.0	10.0000	1.1547	11.547
M	LED-100	6	10	9.5	9.7000	0.8233	8.487
M	LED-200	1	12	9.0	10.7500	2.8324	26.348
M	LED-200	2	8	10.0	9.6250	0.5175	5.377
M	LED-200	3	9	10.0	10.1111	0.6009	5.943
M	LED-200	4	5	9.0	9.4000	0.5477	5.827
M	LED-200	5	9	10.0	9.5556	0.8819	9.229
M	LED-200	6	7	10.0	9.5714	0.5345	5.584

Table S6. Female days to emergence GLMM conditional and dispersion model estimates, including light type, intensity, and their interaction as fixed effects, with replicate as a random effect for the conditional model. Controls are excluded from this model (n = 513).

Variable	Estimate	Standard Error	z-value	p-value
**Conditional Model**				
Light Type	-0.02058	0.02248	0.92	0.360
Intensity	-0.0001309	0.0001517	-0.86	0.388
Light Type x Intensity	-0.00004185	0.0002130	-0.20	0.844
**Dispersion Model**				
**Light Type**	**0.540200**	**0.205615**	**2.627**	**0.00861**
**Intensity**	**-0.003508**	**0.001456**	**-2.409**	**0.01598**
**Light Type x Intensity**	**0.003322**	**0.001954**	**1.700**	**0.08907**

Table S7. Male days to emergence GLMM conditional and dispersion model estimates, including light type, intensity, and their interaction, as fixed effects, with replicate as a random effect for the conditional model. Controls are excluded from this model (n = 498).

Variable	Estimate	Standard Error	z-value	p-value
**Conditional Model**				
Light Type	-0.01278	0.02143	-0.60	0.551
Intensity	-0.000009796	0.0001515	-0.06	0.948
Light Type x Intensity	0.00001482	0.0002093	0.07	0.944
**Dispersion Model**				
Light Type	-0.305498	0.189814	-1.609	0.10752
**Intensity**	**-0.004656**	**0.001223**	**-3.808**	**0.00014**
Light Type x Intensity	0.002647	0.001763	1.501	0.13324

Journal of Experimental Biology • Supplementary information

Table S8. Female days to emergence GLMM conditional and dispersion model estimates with light type as fixed effect with levels of HPS, LED, and Control, and replicate as a random effect for the conditional model (n = 559). Estimated coefficients represent estimated differences from the control group.

Variable	Estimate	Standard Error	z-value	p-value
**Conditional Model**				
HPS	-0.0185558	0.0252844	-0.73	0.463
LED	-0.0005614	0.0252757	-0.02	0.982
**Dispersion Model**				
**HPS**	**0.4906**	**0.2272**	**2.159**	**0.0308**
**LED**	**1.2969**	**0.2275**	**5.701**	**1.19×10^{-8}**

Table S9. Male days to emergence GLMM conditional and dispersion model estimates with light type as fixed effect with levels of HPS, LED, and Control, and replicate as a random effect for the conditional model (n = 555). Estimated coefficients represent estimated differences from the control group.

Variable	Estimate	Standard Error	z-value	p-value
**Conditional Model**				
HPS	-0.005797	0.021801	-0.27	0.790
LED	-0.017557	0.021814	-0.80	0.421
**Dispersion Model**				
**HPS**	**0.8078**	**0.2100**	**3.847**	**0.00012**
**LED**	**0.6507**	**0.2097**	**3.103**	**0.00192**

Table S10. Female days to emergence GLMM conditional and dispersion model estimates with treatment (combining light type and intensity categories) as a fixed effect and replicate as a random effect for the conditional model (n = 559). Estimated coefficients represent estimated differences from the control group.

Variable	Estimate	Standard Error	z-value	p-value
**Conditional Model**				
HPS-10	-0.017154	0.032889	-0.52	0.602
HPS-30	-0.001875	0.031900	-0.06	0.953
HPS-60	-0.025240	0.032596	-0.77	0.439
HPS-100	-0.012453	0.030556	-0.41	0.684
HPS-200	-0.036710	0.031614	-1.16	0.246
LED-10	-0.026947	0.031166	-0.86	0.387
LED-30	0.052650	0.032154	1.64	0.102
LED-60	0.022908	0.031441	0.73	0.466
LED-100	-0.024689	0.032083	-0.77	0.442
LED-200	-0.022155	0.031920	-0.69	0.488
**Dispersion Model**				
**HPS-10**	**0.5514**	**0.2985**	**1.847**	**0.06469**
**HPS-30**	**0.6766**	**0.2907**	**2.328**	**0.01992**
HPS-60	0.1531	0.2944	0.520	0.60311
**HPS-100**	**0.8225**	**0.2782**	**2.956**	**0.00312**
HPS-200	-0.1586	0.2906	-0.546	0.58527
**LED-10**	**0.5024**	**0.2824**	**1.779**	**0.07523**
**LED-30**	**2.0333**	**0.3004**	**6.768**	**1.31×10^{-11}**
**LED-60**	**1.2091**	**0.2885**	**4.190**	**2.78×10^{-5}**
LED-100	0.4681	0.2908	1.610	0.10744
**LED-200**	**1.4157**	**0.2903**	**4.877**	**1.08×10^{-6}**

Journal of Experimental Biology • Supplementary information

Table S11. Male days to emergence GLMM conditional and dispersion model estimates with treatment (combining light type and intensity categories) as a fixed effect and replicate as a random effect for the conditional model (n = 555). Estimated coefficients represent estimated differences from the control group.

Variable	Estimate	Standard Error	z-value	p-value
**Conditional Model**				
HPS-10	0.015058	0.028150	0.53	0.593
HPS-30	-0.027812	0.028337	-0.98	0.326
HPS-60	0.017455	0.028402	0.61	0.539
HPS-100	-0.043427	0.029396	-1.48	0.140
HPS-200	0.007545	0.029431	0.26	0.798
LED-10	-0.016654	0.029768	-0.56	0.576
LED-30	-0.008889	0.028098	-0.32	0.752
LED-60	-0.031191	0.029152	-1.07	0.285
LED-100	-0.018499	0.027992	-0.66	0.509
LED-200	-0.014231	0.028666	-0.50	0.620
**Dispersion Model**				
**HPS-10**	**1.6084**	**0.2745**	**5.859**	**4.65×10^{-9}**
**HPS-30**	**-0.6456**	**0.2720**	**5.859**	**0.0176**
**HPS-60**	**1.1821**	**0.2765**	**4.276**	**1.91×10^{-5}**
**HPS-100**	**-1.1674**	**0.2901**	**-4.024**	**5.71×10^{-5}**
HPS-200	0.4565	0.2834	1.611	0.1073
**LED-10**	**0.6303**	**0.2855**	**2.208**	**0.0273**
**LED-30**	**1.2532**	**0.2711**	**4.623**	**3.79×10^{-6}**
LED-60	-0.2534	0.2783	-0.910	0.3626
LED-100	0.3706	0.2686	1.380	0.1677
**LED-200**	**0.5697**	**0.2755**	**2.068**	**0.0387**

Table S12. Output of logistic regression with binomial distribution using glmmTMB on the relationship between survival and treatment group. Each estimate is compared to the controls.

Treatment	Light	Intensity	n	Estimate	SE	z-value	p-value
HPS-10	HPS	10	101	0.6331	0.6285	1.007	0.314
HPS-30	HPS	30	108	0.4700	0.5869	0.801	0.423
HPS-60	HPS	60	109	-0.4655	0.4779	-0.974	0.330
HPS-100	HPS	100	112	0.7405	0.6277	1.180	0.238
HPS-200	HPS	200	108	-0.1574	0.5059	-0.311	0.756
LED-10	LED	10	108	0.1139	0.5362	0.212	0.832
LED-30	LED	30	105	0.4404	0.5871	0.750	0.453
LED-60	LED	60	107	0.1040	0.5363	0.194	0.846
LED-100	LED	100	109	0.7124	0.6279	1.135	0.257
LED-200	LED	200	106	0.2581	0.5580	0.463	0.644

Journal of Experimental Biology • Supplementary information
